# Genome sequencing, assembly, and annotation of the self-flocculating microalga *Scenedesmus obliquus* AS-6-11

**DOI:** 10.1186/s12864-020-07142-4

**Published:** 2020-10-27

**Authors:** Bai-Ling Chen, Wuttichai Mhuantong, Shih-Hsin Ho, Jo-Shu Chang, Xin-Qing Zhao, Feng-Wu Bai

**Affiliations:** 1 State Key Laboratory of Microbial Metabolism, Joint International Research Laboratory of Metabolic & Developmental Sciences, School of Life Sciences and Biotechnology, Shanghai Jiao Tong University, Shanghai, 200240 China; 2 Enzyme Technology Laboratory, National Center for Genetic Engineering and Biotechnology, Pathum Thani, 12120 Thailand; 3 State Key Laboratory of Urban Water Resource and Environment, Harbin Institute of Technology, Harbin, 150090 China; 4 Department of Chemical and Materials Engineering, College of Engineering, Tunghai University, Taichung City, Taiwan; 5 Research Center for Smart Sustainable Circular Economy, Tunghai University, Taichung City, Taiwan; 6 Department of Chemical Engineering, National Cheng Kung University, Tainan City, Taiwan

**Keywords:** Green microalgae, *Scenedesmus obliquus*, Genome assembly and annotation, Comparative genomics, Cell self-flocculation

## Abstract

**Background:**

*Scenedesmus obliquus* belongs to green microalgae and is widely used in aquaculture as feed, which is also explored for lipid production and bioremediation. However, genomic studies of this microalga have been very limited. Cell self-flocculation of microalgal cells can be used as a simple and economic method for harvesting biomass, and it is of great importance to perform genome-scale studies for the self-flocculating *S. obliquus* strains to promote their biotechnological applications.

**Results:**

We employed the Pacific Biosciences sequencing platform for sequencing the genome of the self-flocculating microalga *S. obliquus* AS-6-11, and used the MECAT software for de novo genome assembly. The estimated genome size of *S. obliquus* AS-6-11 is 172.3 Mbp with an N50 of 94,410 bp, and 31,964 protein-coding genes were identified. Gene Ontology (GO) and KEGG pathway analyses revealed 65 GO terms and 428 biosynthetic pathways. Comparing to the genome sequences of the well-studied green microalgae *Chlamydomonas reinhardtii, Chlorella variabilis*, *Volvox carteri* and *Micractinium conductrix*, the genome of *S. obliquus* AS-6-11 encodes more unique proteins, including one gene that encodes D-mannose binding lectin. Genes encoding the glycosylphosphatidylinositol (GPI)-anchored cell wall proteins, and proteins with fasciclin domains that are commonly found in cell wall proteins might be responsible for the self-flocculating phenotype, and were analyzed in detail. Four genes encoding both GPI-anchored cell wall proteins and fasciclin domain proteins are the most interesting targets for further studies.

**Conclusions:**

The genome sequence of the self-flocculating microalgal *S. obliquus* AS-6-11 was annotated and analyzed. To our best knowledge, this is the first report on the in-depth annotation of the *S. obliquus* genome, and the results will facilitate functional genomic studies and metabolic engineering of this important microalga. The comparative genomic analysis here also provides new insights into the evolution of green microalgae. Furthermore, identification of the potential genes encoding self-flocculating proteins will benefit studies on the molecular mechanism underlying this phenotype for its better control and biotechnological applications as well.

**Supplementary information:**

**Supplementary information** accompanies this paper at 10.1186/s12864-020-07142-4.

## Background

Microalgae are widely studied for producing biofuels and mitigating greenhouse gas emissions [1]. In addition, microalgae are also producers of various high-value biochemicals, such as lipids, proteins, polysaccharides, pigments, vitamins, and antioxidants [2]. For economic bioproduction by microalgae, robust strains and optimized processes are both essential [3]. Genome-scale studies of microalgae can provide in-depth information on intracellular metabolism from a global prospect [4–6], and benefit the development of robust microalgal strains and efficient processes. Therefore, studies on genome sequencing and annotation of microalgae have received increasing attention.

Due to small cell size, negative surface charge and low biomass concentration achieved during photosynthetic autotrophic culture, harvesting microalgae biomass from a large volume of culture medium is a great challenge for their biorefinery [7, 8]. Among various technologies developed for microalgal biomass recovery, gravity sedimentation facilitated by the flocculation of microalgal cells is more economically competitive [8]. On the other hand, some microalgal strains can flocculate or aggregate spontaneously [9–11]. The self-flocculation of microalgal cells enables their harvest without the addition of exogenous flocculants, and biomass harvesting based on the self-flocculation of microalgal cells is more environmentally friendly compared to the flocculation of microalgal cells through physical and chemical methods, or induced by infochemicals from predators [8, 12].

Despite the progress in using cell flocculation of microalgae for biomass recovery, in-depth studies on mechanisms of microalgal cell flocculation are still lacking. So far, microalgal cell wall polysaccharide and other extracellular polymeric substances (EPS) containing sugars and proteins have been identified as flocculating agents [10, 11, 13], but genes involved in the process remain unexplored. Identification of genes involved in microalgal cell self-flocculation is of importance to develop robust strains with controlled flocculation phenotype for microalgal biorefinery, which can be enabled by the advancement of genetic engineering of microalgae [14].

*Scenedesmus* belongs to green microalgae and is useful for lipid and pigment production, wastewater treatment, heavy metal removal and CO_2_ fixation [14–18]. The self-flocculating microalga *S. obliquus* AS-6-1 showing great advantages in microalgal cell harvest and heavy metals adsorption was reported previously [10, 16]. However, genes encoding key protein(s) for the synthesis of flocculating agents in *S. obliquus* are still not clear. To date, the genome sequences of four *Scenedesmus* strains are available (NCBI BioProjects PRJNA498405, PRJNA394817, PRJNA394817 and PRJNA428298), but none of them have been annotated adequately. Three of these genomes lack annotation information, and the annotation of the remaining strain *Scenedesmus* sp. ARA is incomplete due to the large contig numbers of 4727 and a low N50 value of 37,561. Additionally, the three *Scenedesmus* genomes were sequenced by the second-generation sequencing (SGS) technology that might cause bias annotation due to shorter read lengths compared with Pacific Biosciences (Pacbio) sequencing technology [19].

In this study, we sequenced the self-flocculating *S. obliquus* AS-6-11 genome using the Pacbio technology and reported its genome assembly and annotation. We explored the metabolic potential of this microalga, and performed comparative genome analyses with the other four annotated microalgal genomes. We also comprehensively analyzed the cell wall proteins of *S. obliquus* AS-6-11 that might act as the flocculating agents for the self-flocculating phenotype. The knowledge obtained in this work can not only benefit understanding and control of the self-flocculation of microalgal cells, but will also provide insights for further genome-scale studies of *S. obliquus* and other related microalgae to explore their biotechnological potentials.

## Results

### Morphological features and genome assembly

**Fig. 1:**
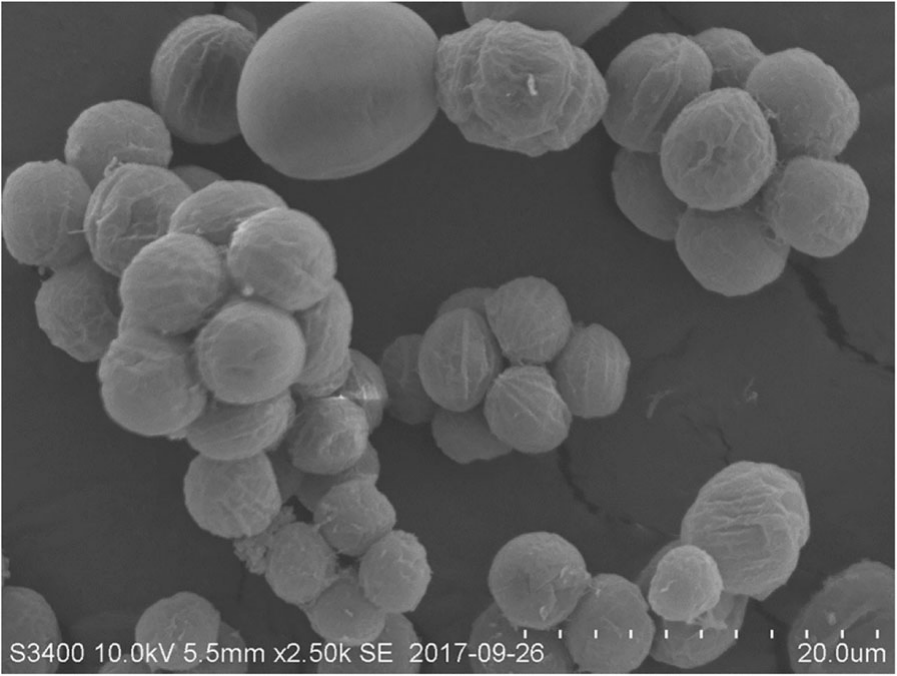
Morphological observation of self-flocculation *S. obliquus* AS-6-11 by SEM

Cell self-flocculation of *S. obliquus* AS-6-11 was observed by SEM analysis. The microalgal cells are round and form aggregates through cell-cell contacts (Fig. 1), which is different from the other reported *Scenedesmus* strains that are in spindle shape [12].

The estimated genome size of *S. obliquus* AS-6-11 is 172.3 Mbp with 2772 contigs, and the N50 contig size is 94.4 kbp using MECAT for the genome assembly (Additional file 1: Table S1; NCBI BioProject ID: PRJNA593662). Results using the MECAT software showed a better assembly ability than that of SMRT Portal in *S. obliquus* AS-6-11, in which the contig numbers are 58.1% less, and the N50 value is 1.5-fold higher (Additional file 1: Table S1). The genome sizes of the released *Scenedesmus* strains [20–24] range from 23.4 to 208.0 Mbp (Table 1). Among the available results, the N50 contig sizes of *S. obliquus* AS-6-11 reported in this study and *S. obliquus* strain DOE0152z using Pacbio technology are significantly higher than the other *Scenedesmus* strains using SGS (Table 1). The N50 contig size of *S. obliquus* AS-6-11 is 1.2-fold and 10.7-fold higher than *Scenedesmus* sp. MC-1 and *S. quadricauda* LWG 002611, respectively. Besides, the GC content of *Scenedesmus* strains ranges from 52.0 to 63.2%, and *S. obliquus* AS-6-11 has the lowest GC content (Table 1). Benchmarking Universal Single-Copy Orthologs (BUSCO) analysis showed that the assembly of *S. obliquus* AS-6-11 is 87.1% complete with 2168 BUSCO groups (Additional file 2).

**Table 1:** Genomic information of the reported *Scenedesmus* strains^a^

Strains	Genome size (Mbp)	GC content (%)	Contig numbers	N50 value (bp)	Sequencing technology	Gene number	Reference/ BioProjects
*Scenedesmus* sp. ARA	93.2	56.8	4727	37,561	Illumina HiSeq	–	[20]
*Scenedesmus* sp. MC-1	38.2	61.4	–	42,815	Illumina HiSeq 2000	8652	[21]
*S. vacuolatus*	23.4	53.6	20,139	1571	454	20,139	PRJNA498405
*S. quadricauda* isolate LWG 002611	65.4	63.2	13,425	8094	Ion Proton	13,514	[22]
*Tetradesmus obliquus* UTEX393	108.7	56.8	9191	–	Illumina Hiseq2000	–	[23]
*S. obliquus* strain DOE0152z	208.0	56.7	2705	155,544	PacBio	–	[24]
*S. obliquus* AS-6-11	172.3	52.0	2772	94,410	PacBio	31,964	This study

^a^- means information not available

### Genome annotations

A total of 31,964 protein-coding genes were predicted in the *S. obliquus* AS-6-11 genome (Table 2). The predicted gene number of *S. obliquus* AS-6-11 genome is dramatically higher than the other *Scenedesmus* strains (Table 1). According to the Non-redundant protein (NR), SWISS-PROT, and Pfam protein families databases, 19,847, 13,099, and 13,612 proteins were annotated, respectively (Table 2). The protein number annotated based on the NR database is the largest, which is 1.52-fold higher than that obtained based on the SWISS-PROT database. Besides, 65 GO terms and 428 pathways were predicted by Gene Ontology (GO) and Kyoto Encyclopedia of Genes and Genomes (KEGG) databases in *S. obliquus* AS-6-11, respectively.

**Table 2:** Summary of the *S. obliquus* AS-6-11 genome annotation

Protein database	Annotated protein numbers
NR	19,847
SWISS-PROT	13,099
Pfam	13,612
GO	11,734
KEGG	3302

**Fig. 2:**
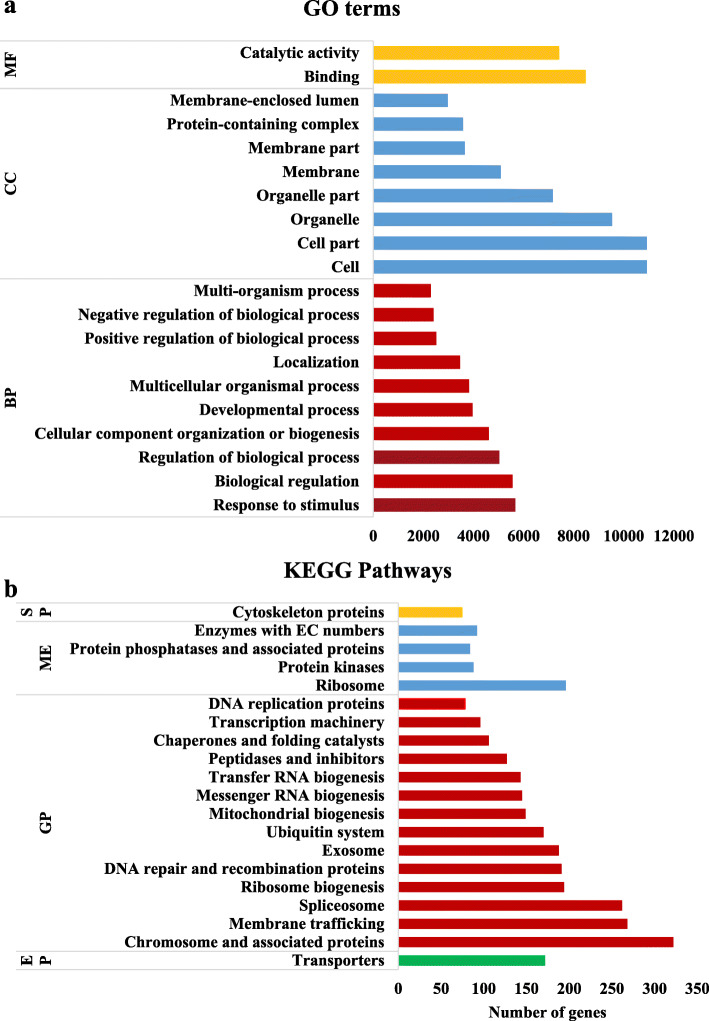
Gene functional annotation analysis of *S. obliquus* AS-6-11 genome. **a** The top 20 of GO categories enriched by gene functional annotation analysis of *S. obliquus* AS-6-11 genome; **b** The top 20 of KEGG pathways enriched by gene functional annotation analysis of *S. obliquus* AS-6-11 genome. BP: Biological process; CC: Cellular component; EP: Environmental information processing; GP: Genetic information processing; ME: Metabolism; MF: Molecular function; SP: Signaling and cellular processes

The top 20 GO terms and KEGG pathways enriched in gene function annotation of the *S. obliquus* AS-6-11 genome were illustrated in Fig. 2. The top 20 GO terms are mainly located in biological process (10) and cellular component (8), in which the cell, cell part, and organelle are the top three GO terms (Fig. 2a). The top 20 KEGG pathways are mainly related to genetic information processing (14), in which chromosome and associated proteins, membrane trafficking, and spliceosome are the top three KEGG pathways (Fig. 2b).

### Comparative genomic analysis based on KEGG pathways

A total of 428 pathways were annotated in the *S. obliquus* AS-6-11 genome. In terms of lipid metabolism, the fewest genes (171) were annotated in *S. obliquus* AS-6-11, especially in glycerolipid metabolism, glycerophospholipid metabolism and arachidonic acid metabolism (Table 3). However, more genes related to fatty acid biosynthesis and elongation were identified in *S. obliquus* AS-6-11 than that in *C. reinhardtii* and *V. carteri* (Table 3). Moreover, genes in the carotenoid biosynthesis in *S. obliquus* AS-6-11 are the fewest.

**Table 3:** Analysis of gene numbers of the key metabolic pathways among the five microalgae

KEGG pathways	*C. reinhardtii*	*C. variabilis*	*M. conductrix*	*V. carteri*	*S. obliquus* AS-6-11
**Lipid metabolism**					
Fatty acid biosynthesis	23	26	27	24	26
Fatty acid elongation	7	8	10	8	8
Fatty acid degradation	16	21	15	18	18
Steroid biosynthesis	9	12	14	9	10
Steroid hormone biosynthesis	5	4	4	4	3
Glycerolipid metabolism	28	28	30	28	21
Glycerophospholipid metabolism	35	37	35	32	30
Ether lipid metabolism	5	9	7	6	5
Sphingolipid metabolism	18	16	14	16	17
Arachidonic acid metabolism	14	13	13	10	7
Alpha-linolenic acid metabolism	10	13	14	11	9
Biosynthesis of unsaturated fatty acids	10	15	14	12	11
**Metabolism of terpenoids and polyketides**					
Carotenoid biosynthesis	12	11	14	12	10

### Comparative genomic analysis of orthologous gene clusters

**Fig. 3:**
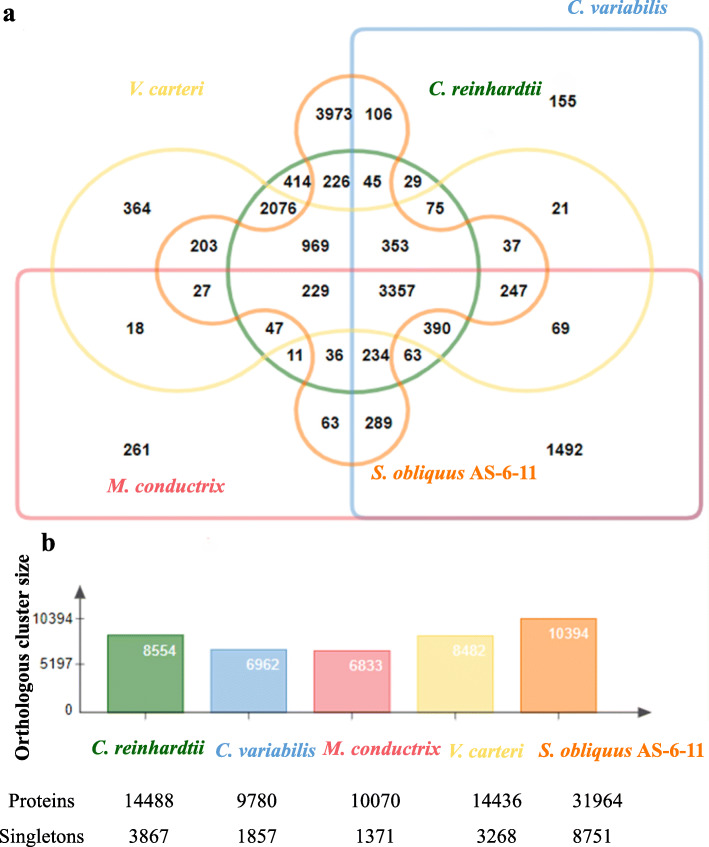
The orthologous gene clusters in the five microalgae. a. The shared and different orthologous gene clusters; b. The size of each orthologous gene clusters

Comparing with the other four species, *S. obliquus* AS-6-11 has 15,879 gene clusters with 14,576 orthologous clusters and 1303 single-copy gene clusters (Fig. 3). There are 3357 overlapping orthologous gene clusters among the five microalgae. *S. obliquus* AS-6-11 has the most gene clusters and singletons (defined as the singleton genes for which no orthologs could be found in any of the other species [25]), and the number (8751) is 1.26-fold, 3.71-fold, 5.34-fold and 1.67-fold higher than that in *C. reinhardtii*, *C. variabilis*, *M. conductrix* and *V. carteri*, respectively (Fig. 3)*.* Comparative orthologous gene cluster analysis also showed that the phylogenetic proximity of *S. obliquus* AS-6-11 is very similar to that of the other four microalgae (Additional file 3: Fig. S1).

### Comparative genomic analysis based on gene families

**Fig. 4:**
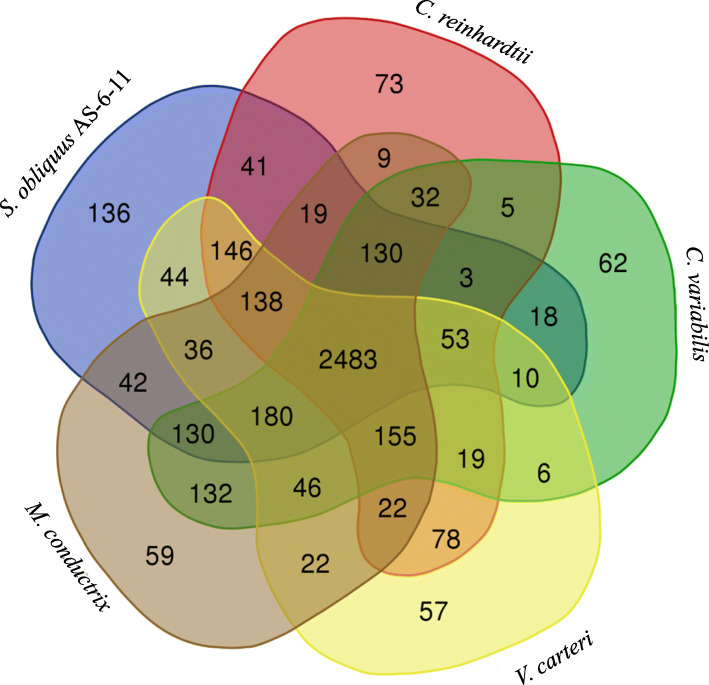
Venn diagram of shared/unique gene families among the five microalgae

A total of 3608 gene families were identified in *S. obliquus* AS-6-11, in which 136 unique gene families existed (Fig. 4). Both the total and unique gene families in *S. obliquus* AS-6-11 are more abundant than that in the other four microalgae (Fig. 4). The number of the unique gene families in *S. obliquus* AS-6-11 is 0.86, 1.19, 1.31 and 1.39-fold larger than *C. reinhardtii*, *C. variabilis*, *M. conductrix* and *V. carteri*, respectively (Fig. 4)*.* In the *S. obliquus* AS-6-11 genome, the unique gene families include membrane protein (PF10160), red chlorophyll catabolite reductase (RCC reductase, PF06405), D-mannose binding lectin (PF01453), lipase maturation factor (PF06762), lipid-A-disaccharide synthetase (PF02684), thioesterase-like superfamily (PF13279) and so on. In addition, *S. obliquus* AS-6-11 and *M. conductrix* have the most common gene families (Fig. 4).

### Analysis of the genome features related to cell self-flocculation

Cell self-flocculation of budding yeast *Saccharomyces cerevisiae* has been well-studied. The flocculation proteins, for example, Flo1p, Flo5p, Flo9p, and Flo10p, are cell wall proteins (CWPs) and also called lectin [26, 27]. GPI-anchor was reported as the common element in cell adhesion proteins and the GPI-anchored adhesins in yeast species of *Candida albicans* and *S. cerevisiae* are the well-known fungal adhesions [28]. In *S. obliquus* AS-6-11, a total of 432 GPI-anchored CWPs are identified. Analysis of the top 10 GPI-anchored CWPs indicated that seven of them has the transmembrane region, and eight of them had the signal peptides (Table 4). The isoelectric point (pI) and molecular weight (Mw) of the GPI-anchored CWPs vary from 4.95 to 9.58 and 6.10 KDa to 78.84 KDa, respectively (Table 4).

**Table 4:** Analysis of the top 10 GPI-anchored CWPs with signal peptides^a^

Protein name	GPI probability (%)	pI	Mw (KDa)	SMART analysis	Subcellular localization sites
Sco00011036	99.82	6.75	7.13	TMR	vacu: 8, chlo: 2, plas: 2, extr: 1, golg: 1
Sco00023226	99.73	5.38	30.84	TMR	vacu: 8, plas: 4, extr: 2
Sco00002357	99.65	6.36	9.68	TMR	extr: 7, E.R.: 3.5, E.R._plas: 3, mito: 2, plas: 1.5
Sco00003994	99.51	4.95	21.28	–	extr: 12, mito: 1, E.R.: 1
Sco00022819	99.47	8.59	29.73	TMR	extr: 11, mito: 2, vacu: 1
Sco00000470	99.41	9.58	14.83	TMR	vacu: 7, plas: 3, extr: 2, E.R.: 1, golg: 1
Sco00000669	99.33	8.48	6.10	–	extr: 12, mito: 1, plas: 1
Sco00004618	99.02	7.51	15.28	TMR	extr: 9, vacu: 3, chlo: 2
Sco00003952	98.73	7.51	78.84	TMR	plas: 11, vacu: 2, E.R.: 1
Sco00008125	98.71	5.22	8.87	–	plas: 11, extr: 11, vacu: 2, nucl: 1, cyto: 1, E.R.: 1

^a^*chlo* chloroplast, *cyto* cytoplasmic, *E.R.* endoplasmic reticulum, *extr* secreted, *golg* golgi apparatus, *mito* mitochondrial matrix, *plas* membrane protein, *TMR* Transmembrane region, *vacu* vacuolar. ‘-’ represented no information available

**Fig. 5:**
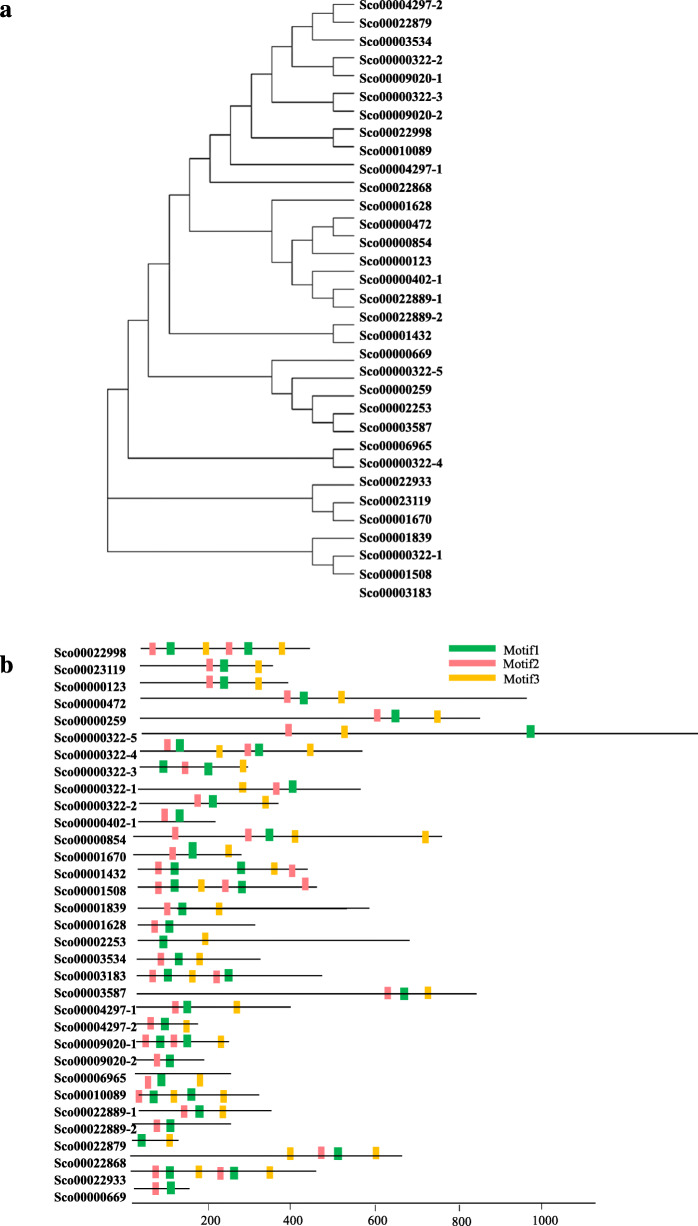
The phylogenetic tree (**a**) and conserved motifs (**b**) of the fasciclin domain proteins in *S. obliquus* AS-6-11

Fasciclin (PF02469) is an extracellular domain (http://pfam.xfam.org/family/PF02469) that belongs to the ancient cell adhesion domain that is common to plants and animals. So far, fasciclin domain proteins have not been analyzed in microalgae. In the *S. obliquus* AS-6-11 genome, a total of 33 fasciclin domain proteins are identified, which are divided into three groups (Fig. 5a). Three main motifs are randomly distributed across the fasciclin domain proteins (Fig. 5b). The predicted pI values and Mw greatly differ among the fasciclin domain proteins (Additional file 4: Table S2). The subcellular localization prediction of fasciclin domain proteins indicated that most proteins have cytoplasmic (cyto) sites, and 15 of them have secreted (extr) sites (Additional file 4: Table S2). Further analysis of these 15 fasciclin domain proteins containing extr sites showed that six proteins are homologous to the reported fasciclin proteins of *Monoraphidium neglectum* (64.84%), *Aquabacterium* sp. (61.36%), *Scenedesmus* sp. Ki4 (48.09%), *Pelomonas puraquae* (46.94%) (Table 5). Additionally, two of the predicted proteins are annotated into the extracellular region part according to the GO database.

**Table 5:** Analysis of predicted extracellular secreted fasciclin domain proteins in *S. obliquus* AS-6-11^a^

Protein name	pI	Mw (KDa)	Signal peptide	The most similar homologous protein and the source organism	Identity to the most similar sequence
Sco00000123	7.5	32.5	–	hypothetical protein MNEG_1104 [*Monoraphidium neglectum*]	63.00%
Sco00000322–1	9.2	43.4	1	hypothetical protein A1O9_09854 [*Exophiala aquamarina* CBS 119918]	40.00%
Sco00000322–2	8.8	35.1	1	fasciclin domain-containing protein [*Aquabacterium* sp.]	61.36%
Sco00000402–1	8.9	23.4	1	hypothetical protein DI09_43p180 [*Mitosporidium daphniae*]	38.78%
Sco00001432	7.7	44.1	1	Nex18 symbiotically induced [*Micractinium conductrix*]	52.08%
Sco00002253	7.1	80.0	–	hypothetical protein MNEG_2497 [*Monoraphidium neglectum*]	46.00%
Sco00003534	4.2	29.9	1	astaxanthin binding fasciclin family protein [*Scenedesmus* sp. Ki4]	48.09%
Sco00003587	7.6	84.4	1	–	
Sco00004297	6.3	16.4	–	hypothetical protein Rsub_06992 [*Raphidocelis subcapitata*]	61.54%
Sco00009020	8.9	18.3	–	fasciclin domain-containing protein [*Aquabacterium* sp.]	61.36%
**Sco00022889–1**	8.9	33.8	1	fasciclin-like protein [*Chlamydomonas reinhardtii*]	38.75%
**Sco00022889–2**	7.7	26.7	1	fasciclin [*Pelomonas puraquae*]	46.94%
Sco00022879	5.2	12.2	–	beta-Ig-H3/fasciclin [*Monoraphidium neglectum*]	64.84%
Sco00000669	4.9	14.5	–	fasciclin domain-containing protein [*Marinobacter*]	53.85%

^a^The protein-coding genes that encode GPI-anchored CWPs were shown in bold font; ‘-’ represented no information available

**Fig. 6:**
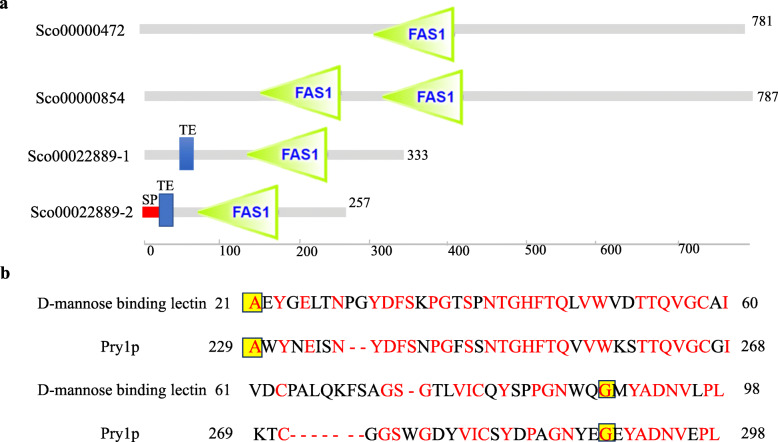
Amino acid analysis of potential flocculation proteins in *S. obliquus* AS-6-11. a. Amino acid analysis of the four fasciclin domain proteins distributed in GPI-anchored CWPs; b. Amino acid differences of CAP domain between partial *S. cerevisiae* Pry1p and *S. obliquus* AS-6-11 D-mannose binding lectin. The same amino acids in Fig. 6 b were shown in red font and the amino acid using the yellow background represented the start/stop of the CAP domain. CAP domain: cysteine-rich secretory proteins domain; FAS1: Four repeated domains in the fasciclin I family of proteins; SP: Signal peptide; TE: Transmembrane region

Combining analysis of GPI-anchored CWPs and fasciclin domain proteins, four fasciclin domain proteins were found to distribute in GPI-anchored CWPs (Fig. 6a; Additional file 5), in which one has two FAS1 domains (four repeated domains in the fasciclin I family of proteins), two have transmembrane regions, and one has signal peptide (Fig. 6a). Comparative genomic analysis of *S. obliquus* AS-6-11 and the other four microalgae species (*C. reinhardtii*, *C. variabilis*, *M. conductrix* and *V. carteri*) revealed no similar proteins to the four fasciclin domain proteins. We also performed comparative transcriptome analysis of *S. obliquus* AS-6-11 and the non-flocculating *S. obliquus* FSP-3, and the results showed that the four fasciclin domain protein-encoding genes (Fig. 6a) had transcription level in *S. obliquus* AS-6-11, but the transcription of these genes cannot be detected in *S. obliquus* FSP-3 (Additional file 6: Table S3).

The unique gene family D-mannose binding lectin was also analyzed (Additional file 5). One gene belongs to this unique gene family was identified, and the encoded protein has two conserved domains: CAP (cysteine-rich secretory proteins) domain and B_lectin (D-mannose binding lectin) domain. The putative D-mannose binding lectin of *S. obliquus* AS-6-11 is homologous to a secreted glycoprotein Pry1p of *S. cerevisiae* YJM693 (SGD ID: S000003615), and the identity is 58% (Fig. 6b). The similarity between Pry1p and D-mannose binding lectin attributes to the same CAP domain (Fig. 6b).

## Discussion

### Genome feature of *S. obliquus* AS-6-11

We report here the genome sequence and annotation of *S. obliquus* AS-6-11, which is the first sequenced self-flocculating microalgal genome, and is also so far the most comprehensively annotated genome among the available *S. obliquus* genome information. The much larger gene numbers of *S. obliquus* AS-6-11 (Table 1) suggested its unique feature. Considering the relative completeness of *S. obliquus* AS-6-11 genome and the lack of *S. obliquus* genome annotation, *S. obliquus* AS-6-11 may serve as a model alga for supplying reference genome annotation and investigating the gene function, evolution, and biotechnology application of *S. obliquus* strains.

We obtained a larger contig N50 size of *S. obliquus* AS-6-11 genome than that of *Scenedesmus* sp. MC- and *S. quadricauda*, which may attribute to Pacbio sequencing technology and the assembly tool MECAT [29]. The MECAT software showed higher computing efficiency with comparable or improved genome results than other current tools for de novo assembly of large genomes [29], and this study is the first application of this tool in the genome assembly of microalgae.

### Comparative analysis of *S. obliquus* AS-6-11 genome with the other microalgae

Due to the lack of genome annotations of *Scenedesmus* strains, we compared the *S. obliquus* AS-6-11 genome with four represented green microalgae (Fig. 3; Fig. 4; Table 3). *S. obliquus* AS-6-11 has more singletons, unique gene families as well as additional KEGG pathways than the other four microalgae, revealing its special evolutionary status, genomic complexity, and metabolic characteristics. Comparative genomics analysis of orthologous clusters among multiple species is important for identifying the overlap among orthologous clusters that helps to elucidate the evolution and function of proteins [25]. The similar phylogenetic proximity of *S. obliquus* AS-6-11 to the four green microalgae further supports that this species locates in the intermediate stage of green algae evolution [30].

Due to the potential applications of *Scenedesmus* strains in wastewater treatment and lipid production [31, 32], their lipid metabolism needs to be well-studied. *S. obliquus* AS-6-11 has fewer genes of lipid metabolism compared to *S. quadricauda* LWG002611 and the other four green microalgae, suggesting the difference of lipid biosynthesis or genome integrity among the strains. For example, the fewer genes of arachidonic acid metabolism in *S. obliquus* AS-6-11 may attribute to multiple genes encoding one enzyme in other microalgae (Table 3). Key enzymes involved in triacylglycerol (TAG) biosynthesis pathways and carbon fixation were identified based on the genome sequence of *S. quadricauda* LWG002611, providing targets for genetic and metabolic engineering to improve biofuel production and reconstruct the metabolic pathways of this strain [22]. In the previous work, overexpression of type 2 diacylglycerol acyltransferase gene (*DGTT1*) of *C. reinhardtii* in *S. obliquus* CPC2 enhanced its lipid content by nearly two-fold [33]. Overexpression of acetyl-CoA carboxylase gene in *Scenedesmus* sp. MC-1 increased its intracellular lipid by 28.6%, indicating its importance in the lipid metabolism of *Scenedesmus* [21]. However, the endogenous genes involved in lipid biosynthesis in *S. obliquus* has not been investigated. The identified lipid biosynthetic genes in this work will facilitate improving lipid production in *S. obliquus*.

On the other hand, *Scenedesmus* can produce important pigments carotenoids as valuable products, and overexpression of synthetic phytoene synthase gene (*PSY*) in *Scenedesmus* sp. CPC2 increased β-carotene production to 30 mg g^− 1^-cell [34]. So far no studies have been focused on the innate carotenoid biosynthetic genes in *S. obliquus*. Further investigation of the functional genome of *Scenedesmus* will benefit the metabolic engineering of this important microalga for carotenoids production.

### Potential flocculation proteins in *S. obliquus* AS-6-11

Although bioflocculation has been widely accepted as a promising way to harvest microalgal biomass, studies on cell self-flocculation of microalgae are still very limited. In the previous study, cell wall polysaccharides were revealed to act as flocculating agents in *S. obliquus* AS-6-1 [10]. In the recent study, we found that protease treatment led to the de-flocculation of *S. obliquus* AS-6-11 (data not shown), indicating their different mechanisms of cell self-flocculation. Therefore, we focused on the identification of flocculating proteins in *S. obliquus* AS-6-11.

Yeast cell flocculation has been widely used in the beer industry and biofuels production as a simple, economic and environmentally friendly way to harvest cells [35, 36]. The interaction of lectin-like receptors with adjacent mannose side chains in cell walls played a vital role in yeast flocculation [37]. In addition to lectin-glycan interaction, glycan-glycan interactions also act a pivotal part in cell-cell adhesion, and the flocculation mechanism contributes to the self-interaction of Flo proteins in the coordination of Ca^2+^ [38]. In our recent studies, mechanisms of cell flocculation in an ethanol-producing bacterium *Zymomonas mobilis* were also revealed, where cellulose was found to be important for the cell flocculation [39]. Moreover, our previous studies [10, 11] found that cell wall polysaccharides play key roles in microalgal cell self-flocculation. Considering that mannose-specific lectin plays an important role in yeast flocculation [40], the D-mannose binding lectin family gene found in the *S. obliquus* AS-6-11 genome may be related to cell flocculation. However, we did not find the transcription of this gene in the transcriptome data of *S. obliquus* AS-6-11. In the water surface-floating microalga *Chlorococcum* sp. FFG039, one protein in the unique gene family (jacalin-like lectin domain, PF01419) was assumed to be related to biofilm formation with the help of the lectin domain [41]. However, the gene encoding jacalin-like lectin domain protein was not found in the *S. obliquus* AS-6-11 genome, suggesting the different mechanisms of microalgal flocculation.

GPI-anchored proteins are widespread in eukaryotes for anchoring proteins to the extracellular surface of the plasma membrane, and are involved in multiple cellular functions [42]. GPI-anchored CWP Flo1p in *S. cerevisiae* is the main flocculation protein, and the GPI-anchor is necessary for Flo1p to attach to the cell wall [27]. However, the GPI-anchored CWPs in microalgae have not been analyzed. Therefore, the genome-scale analysis of GPI-anchored CWPs is of great importance. In the genome of the model plant *Arabidopsis thaliana,* 210 GPI-anchored proteins were identified, and most of these proteins were involved in the primary modification for targeting specific proteins to the cell surface for extracellular matrix remodeling and signaling [43]. According to the GPI-anchor studies of *S. cerevisiae* and *Arabidopsis*, the identified cell wall proteins with GPI-anchor of *S. obliquus* AS-6-11 may be related to the attachment of proteins to the cell wall, and the potential flocculation proteins may be members among them.

Fasciclin 1 (FAS1) domain is an ancient motif in extracellular proteins widely exists in all kingdoms of life, and FAS1 proteins mediated the interactions between the cell surface and cell exterior [44]. FAS1 domain protein of *V. carteri* that is homology to *Drosophila fasciclin* I was identified as the cell adhesion protein [45], and it also caused unicells of *C. reinhardtii* to flocculate [46]. Therefore, the four proteins identified in this study (Fig. 6a), which are homologous to fasciclin domain-containing proteins of *Synechocystis*, *D. melanogaster* and *Galdieria sulphuraria* or hypothetical protein of *M. neglectum* and *C. variabilis*, are the most likely flocculation proteins. Although we have made great efforts to investigate the potential flocculating gene function by gene disruption, the genetic transformation method of *S. obliquus* AS-6-11 was not successful. Alternatively, the protein-encoding genes can also be tested in various microalgal species, which will be focused in future studies. It needs to point out that in addition to the function of cell adhesion and/or flocculation, the fasciclin domain proteins in microalgae also take parts in stress response and cell wall formation. The gene encoding one fasciclin domain protein (carotenoprotein) in microalga Ki-4 was overexpressed under salt, dehydration and high light stresses, showing its function in protecting cells against photooxidative stresses [47]. In addition, the fasciclin-like arabinogalactan protein family in higher plant *Eucalyptus grandis* took parts in the growth and properties of the secondary cell wall [48]. To the best of our knowledge, this is the first report on the analysis of fascilin domain proteins in microalgae. It will be interesting to further explore the functions of this category of proteins. The in-depth analysis of *S. obliquus* AS-6-11 genome can also provide a basis for functional genomic studies of other microalgae.

## Conclusions

We present here the genome sequencing, annotation, and analysis of the self-flocculating microalga *S. obliquus* AS-6-11. Comparative genomic analysis between *S. obliquus* AS-6-11 and the other microalgae reveals its strain specificities, evolutionary status as well as metabolic characteristics. Through the analysis of the protein family, the GPI-anchored CWPs and fasciclin domain proteins were identified for the first time in microalgae, and four GPI-anchored CWPs with fasciclin domain are the most potential flocculation proteins for further studies. Taken together, the draft genome of *S. obliquus* AS-6-11 will provide a reliable reference for the microalgae genome studies, increasing the understanding of microalgal self-flocculation mechanisms for promoting the microalgal harvest, and benefit efficiency biorefinery using microalgae.

## Methods

### Microalgal strain and culture conditions

The self-flocculating *S. obliquus* AS-6-11 was isolated from the freshwater pond in the campus of National Cheng Kung University (22°99′74.29″N, 120°22′22.30″E) in southern Taiwan, and was preserved at National Cheng Kung University. Cells were cultured at 25 °C in BG11 medium with continuous illumination of 75 μmol m^2^ s^− 1^ and continuous air aeration.

### Morphological observation

The cell shape and surface morphology of *S. obliquus* AS-6-11 cells were observed by scanning electron microscope (SEM, Hitachi S-3400 N II). For SEM, cells during the exponential growth phase were harvested and fixed in 2.5% glutaraldehyde solution overnight. After that, the samples were washed two times using PBS buffer (10 mM, pH 7.4), dehydrated in different concentrations of ethanol solutions, and then referred to the method of Salim et al. [49].

### Genome DNA preparation and quality assessment

Two hundred milliliter cells at the exponential growth phase (Day 6) were harvested. The genome DNA of *S. obliquus* AS-6-11 was extracted by EZ-10 Spin Column Plant Genomic DNA Purification Kit (NO. B518261, Sangon Biotech (Shanghai) Co., Ltd.). The DNA quality was monitored and controlled by Nanodrop (Thermo Scientific NanoDrop 2000) and DNA gel electrophoresis.

### Genome sequencing and assembly

The obtained high-quality DNA was sequenced using the PacBio RS II System. The size of DNA libraries was 10–20 kb and 10 SMRT cells were sequenced. Filtered Pacbio subreads were de novo assembled by software MECAT [29] and SMRT Portal with recommending parameters, respectively. Genome assembly quality using MECAT was further assessed by BUSCO (3.0.2) [50]. The following analyses were based on the MECAT assembly result.

### Genome annotation and comparative transcriptome analysis

MAKER2 training and annotation pipeline [51] with AUGUST [52] and SNAP [53] was used for genome structural annotation of *S. obliquus* AS-6-11 according to the *C. reinhardtii* training set and *S. obliquus* AS-6-11 transcriptome data. The transcriptome sequencing was performed by Illumina Hiseq platform. Functional annotation was performed by BLASTp (2.7.1+) [54] according to NR (https://ftp.ncbi.nlm.nih.gov/blast/db/FASTA/) and Swiss-Prot protein (https://ftp.ncbi.nlm.nih.gov/blast/db/) databases. GO (http://geneontology.org/docs/download-ontology/) and KEGG (http://www.genome.jp/kegg/ko.html) annotations were performed according to the analysis method of Tamanna Sharma and Rajinder Singh Chauhan [17]. Gene family analysis was performed by InterProScan 5.36–75.0-64 [55] using the amino acid sequences of *S. obliquus* AS-6-11, *C. reinhardtii* (NCBI accession number: ABCN00000000.2), *C. variabilis* (NCBI accession number: ADIC00000000.1), *V. carteri* (NCBI accession number: ACJH00000000.1) and *M. conductrix* (NCBI accession number: LHPF00000000.2). In addition, the orthologous gene clusters between these microalgal genomes were compared using OrthoVenn2 (https://orthovenn2.bioinfotoolkits.net/) [25].

For transcriptome analysis, *S. obliquus* AS-6-11 and *S. obliquus* FSP-3 cells grown for 48 h under the same condition were harvested at 6000 rpm for 5 min, and then washed three times with the sterilized water. The washed cells were stored at − 80 °C immediately and send to Novogene Co., Ltd. for initial sequencing and analysis.

### Prediction of GPI-anchored cell wall proteins

GPI-anchored CWPs in *S. obliquus* AS-6-11 were predicted by the GPI-anchored protein predictor developed by the National Science and technology development agency of Thailand [56]. The top 10 of predicted GPI-anchored CWPs with signal peptide were further analyzed using compute pI/Mw tools of ExPASy (https://web.expasy.org/compute_pi/) and SMART (http://smart.embl-heidelberg.de/). Protein subcellular localization sites were estimated by Protein Subcellular Localization Prediction (https://wolfpsort.hgc.jp/).

### Analysis of fasciclin domain proteins

The fasciclin domain family proteins were extracted from protein family annotation files and further analyzed using compute pI/Mw tool of ExPASy, SMART, and Protein Subcellular Localization Prediction.

### Phylogenetic analysis of fasciclin domain proteins

A phylogenetic tree of fasciclin domain proteins was constructed using MEGA 7 [57]. The sequence alignment was performed using MUSCLE and the phylogenetic tree was constructed using the Maximum likelihood method with 1000 bootstrap replicates.

### Analysis of the conserved motifs of fasciclin domain proteins

The motifs’ analysis of fasciclin domain proteins was performed by the online MEME website (http://meme-suite.org/tools/meme) with the default parameters.

### Analysis of conserved domains and homologous protein

The analyses of conserved domains and homologous protein were performed using NCBI CD-Search (https://www.ncbi.nlm.nih.gov/Structure/cdd/wrpsb.cgi) and NCBI-blastp choosing the UniProtKB/Swiss-Prot database (https://blast.ncbi.nlm.nih.gov/Blast.cgi?PROGRAM=blastp&PAGE_TYPE=BlastSearch&LINK_LOC=blasthome), respectively.

## Supplementary information

**Additional file 1: Table S1.** Genomic features of *S. obliquus* AS-6-11 using MECAT and SMRT Portal. (DOCX 14 kb)

**Additional file 2:.** Assessing genome completeness with BUSCO. (TXT 880 bytes)

**Additional file 3: Figure S1.** The pairwise heatmap of the overlapping cluster numbers between the pair-wise genomes. (DOCX 55 kb)

**Additional file 4: Table S2.** Analysis of the fasciclin domain proteins in *S. obliquus* AS-6-11. (DOCX 20 kb)

**Additional file 5: **The amino acid sequences of the potential flocculation proteins of *S. obliquus* AS-6-11. (TXT 2 kb)

**Additional file 6: Table S3.** Cq value for the potential flocculating genes in *S. obliquus* AS-6-11 compared to *S. obliquus* FSP-3. (DOCX 14 kb)

## Data Availability

The genome sequence information of *S. obliquus* AS-6-11 was submitted to NCBI with the accession number of PRJNA593662. The amino acid sequences of the potential flocculation proteins of *S. obliquus* AS-6-11 can be found in Additional file 5. The genome sequences of *C. reinhardtii* (NCBI accession number: ABCN00000000.2), *C. variabilis* (NCBI accession number: ADIC00000000.1), *V. carteri* (NCBI accession number: ACJH00000000.1) and *M. conductrix* (NCBI accession number: LHPF00000000.2) were downloaded from the related websites: https://www.ncbi.nlm.nih.gov/genome/?term=ABCN00000000.2%2C, https://www.ncbi.nlm.nih.gov/genome/?term=ADIC00000000.1, https://www.ncbi.nlm.nih.gov/genome/?term=ACJH00000000.1, and https://www.ncbi.nlm.nih.gov/genome/?term=LHPF00000000.2, respectively.

## Abbreviations

BP: biological process
BUSCO: Benchmarking Universal Single-Copy Orthologs
CC: cellular component
chlo: chloroplast
CWPs: cell wall proteins
cysk: cytoskeleton
cyto: cytoplasmic
*DGTT1*: type 2 diacylglycerol acyltransferase gene
EP: environmental information processing
EPS: extracellular polymeric substances
E.R.: endoplasmic reticulum
extr: secreted
FAS1 domain: four repeated domains in the fasciclin I family of proteins
golg: golgi apparatus
GP: genetic information processing
GPI-anchor: glycosylphosphatidylinositol-anchor
ME: metabolism
MF: molecular function
mito: mitochondrial matrix
Mw: molecular weight
NR: non-redundant
nucl: nuclear
pI: isoelectric point
plas: membrane protein
*PSY*: synthetic phytoene synthase gene
SGS: second-generation sequencing
SP: signaling and cellular processes
vacu: vacuolar
