# Supplementary material for: Genome sequencing, assembly, and annotation of the self-flocculating microalga Scenedesmus obliquus AS-6-11
Source: BMC Genomics. 2020 Oct 27;21:743. doi: 10.1186/s12864-020-07142-4 (PMC7590803; doi:10.1186/s12864-020-07142-4)
Supplement: Supplementary file 1 — Additional file 1: Table S1. Genomic features of S. obliquus AS-6-11 using MECAT and SMRT Portal. (DOCX 14 kb) [file 12864_2020_7142_MOESM1_ESM.docx]

Table S1 Genomic features of *S. obliquus* AS-6-11 using MECAT and SMRT Portal

| Genomic features | MECAT | SMRT Portal |
| --- | --- | --- |
| Total assembly length | 172,339,266 bp | 146,613,032 bp |
| Contig counts | 2,772 | 6,621 |
| Largest contig | 1,807,243 bp | 944,801 bp |
| Contig N50 | 94,410 bp | 37,607 bp |
| Contig N90 | 26,581 bp | 10,523 bp |
| L50 | 448 bp | 1,072 bp |
| L90 | 1,886 bp | 3,945 bp |
| GC content (%) | 52.0 | 52.0 |
