# Supplementary figures and images for: Genome sequencing, assembly, and annotation of the self-flocculating microalga Scenedesmus obliquus AS-6-11
Source: BMC Genomics. 2020 Oct 27;21:743. doi: 10.1186/s12864-020-07142-4 (PMC7590803; doi:10.1186/s12864-020-07142-4)

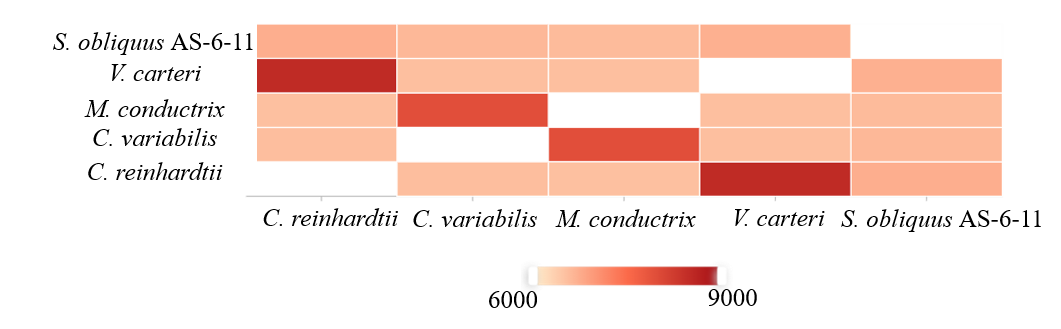


Figure S1 The pairwise heatmap of overlapping cluster numbers between the pair-wise genomes

Supplement: Supplementary file 3 — Additional file 3: Figure S1. The pairwise heatmap of the overlapping cluster numbers between the pair-wise genomes. (DOCX 55 kb) [file 12864_2020_7142_MOESM3_ESM.docx]
