# Supplementary material for: Genome sequencing, assembly, and annotation of the self-flocculating microalga Scenedesmus obliquus AS-6-11
Source: BMC Genomics. 2020 Oct 27;21:743. doi: 10.1186/s12864-020-07142-4 (PMC7590803; doi:10.1186/s12864-020-07142-4)
Supplement: Supplementary file 4 — Additional file 4: Table S2. Analysis of the fasciclin domain proteins in S. obliquus AS-6-11. (DOCX 20 kb) [file 12864_2020_7142_MOESM4_ESM.docx]

Table S2 Analysis of fasciclin domain proteins in *S. obliquus* AS-6-11 genome^*^

| Protein name | pI | Mw (KDa) | Subcellular localization sites | Signal peptide | FAS1 domain (start:end) |
| --- | --- | --- | --- | --- | --- |
| Sco00022998 | 4.45 | 34.51 | cyto: 5, chlo: 4, mito: 2, pero: 2, cysk_nucl: 1 | - | 38:149; 186:299 |
| Sco00023119 | 9.33 | 28.61 | cyto: 7, nucl: 3, cysk: 2, chlo: 1, E.R._vacu: 1 | - | 119:249 |
| Sco00000123 | 7.50 | 32.52 | extr: 8, chlo: 5, mito: 1 | - | 148:260 |
| **Sco00000472** | 6.25 | 79.40 | chlo: 8, nucl: 2, cyto: 1, mito: 1, plas: 1, golg: 1 | - | 296:408 |
| Sco00000259 | 5.81 | 73.95 | nucl: 5, chlo: 3, cyto: 1, mito: 1, plas: 1, vacu: 1, E.R.: 1, golg: 1 | - | 469:591 |
| Sco00000322-1 | 9.23 | 43.44 | vacu: 9, chlo: 3, mito: 1, extr: 1 | 1 | 246:354 |
| Sco00000322-2 | 8.80 | 35.11 | vacu: 6, chlo: 3, extr: 3, nucl: 1, golg: 1 | 1 | 149:324 |
| Sco00000322-3 | 9.65 | 24.67 | chlo: 10, nucl: 2, mito: 2 | - | 39:102; 103:219 |
| Sco00000322-4 | 9.38 | 47.77 | chlo: 7, mito: 5, E.R.: 2 | 1 | 62:180; 220:351 |
| Sco00000322-5 | 7.74 | 114.95 | plas: 11, nucl: 1, vacu: 1, E.R.: 1 | - | 295:412 |
| Sco00000402-1 | 8.86 | 23.36 | extr: 11, vacu: 3 | 1 | 44:149 |
| Table S2 continued | | | | | |
| Sco00000402-2 | 7.92 | 80.08 | chlo: 9, nucl: 1, cyto: 1, mito: 1, plas: 1, golg: 1 | - | 127:248; 302:414 |
| Sco00001670 | 9.85 | 26.93 | chlo: 7.5, chlo_mito: 7.5, mito: 6.5 | - | 93:229 |
| Sco00001432 | 7.65 | 44.08 | vacu: 5, chlo: 4, extr: 2, nucl: 1, mito: 1, golg: 1 | 1 | 227:342 |
| Sco00001508 | 8.94 | 47.26 | plas: 10, E.R.: 2, vacu: 1, golg: 1 | 1 | 57:169; 210:323 |
| Sco00001839 | 9.72 | 62.46 | chlo: 6, mito: 6, nucl: 2 | - | 93:229 |
| Sco00001628 | 8.11 | 30.79 | chlo: 6, cyto: 5, nucl: 2, mito: 1 | - | 57:161 |
| Sco00002253 | 7.05 | 70.97 | plas: 5, E.R.: 5, vacu: 2, nucl: 1, extr: 1 | - | 79:191 |
| Sco00003534 | 4.23 | 29.87 | chlo: 8, extr: 2, vacu: 2, cyto: 1, mito: 1 | 1 | 72:182 |
| Sco00003183 | 8.57 | 47.06 | plas: 9, E.R.: 3, vacu: 1, golg: 1 | 1 | 57:169; 210:323 |
| Sco00003587 | 7.57 | 84.36 | extr: 7, golg: 2, E.R.: 1.5, E.R._plas: 1.5, chlo: 1, cyto: 1, mito: 1 | 1 | 627:739 |
| Sco00004297-1 | 5.43 | 40.74 | cyto: 8, nucl: 3, chlo: 2, mito: 1 | - | 104:268 |
| Sco00004297-2 | 6.27 | 16.41 | mito: 5, cyto: 4, chlo: 3, plas: 1, extr: 1 | - | 43:149 |
| Sco00009020-1 | 10.38 | 23.94 | chlo: 10, mito: 4 | - | 13:102; 103:219 |
| Sco00009020-2 | 8.90 | 18.32 | cyto: 7, chlo: 5, nucl: 1, extr: 1 | - | 58:160 |
| Table S2 continued | | | | | |
| **Sco00022889-1** | 8.87 | 33.77 | chlo: 5, plas: 4, extr: 3, E.R.: 2 | [Transmembrane region](javascript:domWin(3)) | 125:225 |
| Sco00006965 | 4.93 | 23.62 | chlo: 5, mito: 4, cyto: 2, nucl: 1.5,  cysk_nucl: 1.5, plas: 1 | - | 35:170 |
| Sco00010089 | 4.12 | 27.00 | cyto: 9, chlo: 2, nucl: 1, mito: 1, plas: 1 | - | 13:124; 129:227 |
| **Sco00022889-2** | 7.68 | 26.67 | chlo: 5, extr: 3, mito: 2, vacu: 2, nucl: 1, golg: 1 | 1 | 47:152 |
| Sco00022879 | 5.16 | 12.22 | extr: 8, vacu: 5, chlo: 1 | - | 18:113 |
| Sco00022868 | 5.80 | 71.62 | mito: 10, chlo: 3, cyto: 1 | - | 474:615 |
| **Sco00000854** | 8.69 | 44.17 | chlo: 10, cyto: 2, mito: 2 |  | 127:248; 302: 411 |
| Sco00022933 | 9.25 | 47.51 | chlo: 8, mito: 4, E.R.: 2 | 1 | 62:180; 220:351 |
| Sco00000669 | 4.94 | 14.46 | extr: 6, vacu: 3, chlo: 2, nucl: 1, E.R.: 1, golg: 1 | - | 20:131 |

^*^The fasciclin domain proteins that existed in GPI-anchored CWPs were shown in bold font. ‘-’ represented no information available. chlo: chloroplast; cysk: cytoskeleton; cyto: cytoplasmic; E.R.: endoplasmic reticulum; extr: secreted; golg: golgi apparatus; mito: mitochondrial matrix; nucl: nuclear; plas: membrane protein; per: glyoxysomal; vacu: vacuolar.
