# Supplementary material for: Genome sequencing, assembly, and annotation of the self-flocculating microalga Scenedesmus obliquus AS-6-11
Source: BMC Genomics. 2020 Oct 27;21:743. doi: 10.1186/s12864-020-07142-4 (PMC7590803; doi:10.1186/s12864-020-07142-4)
Supplement: Supplementary file 6 — Additional file 6: Table S3. Cq value for the potential flocculating genes in S. obliquus AS-6-11 compared to S. obliquus FSP-3. (DOCX 14 kb) [file 12864_2020_7142_MOESM6_ESM.docx]

Table S3 Cq value for the potential flocculating genes in *S. obliquus* AS-6-11 compared to *S. obliquus* FSP-3^*^

| Gene Name | Cq (*S. obliquus* AS-6-11) | Cq (*S. obliquus* FSP-3) |
| --- | --- | --- |
| Sco00000472 | 34.55 | 0 |
| Sco00000854 | 34.55 | 0 |
| Sco00022889-1 | 31.57 | 0 |
| Sco00022889-2 | 31.14 | 0 |

^*^Cq value means cycle quantification value.
